# A Modified Temperature-Dependent Elastoplastic Constitutive Model for Blister Growth in Irradiated Al6061 Cladding

**DOI:** 10.3390/ma19153233

**Published:** 2026-07-30

**Authors:** Qi Luo, Wei Wang, Yuqing Chen, Fulong Tang

**Affiliations:** College of Nuclear Science and Technology, Naval University of Engineering, No. 717 Jiefang Avenue, Wuhan 430033, China; m24182703@nue.edu.cn (Q.L.); chenyuqing301@163.com (Y.C.); d26382701@nue.edu.cn (F.T.)

**Keywords:** plate-type fuel element blistering, plastic evolution, temperature softening, finite element method, irradiation damage

## Abstract

Plate-type fuel elements generate large amounts of fission gases under irradiation, and the resulting cladding blistering caused by fission gas accumulation compromises both fuel integrity and nuclear reactor safety. Up to now, there appears to be insufficient understanding of blister growth behavior under varying temperature conditions. Therefore, for increased accuracy and safety, we develop a modified quasi-static finite-element model incorporating a bilinear isotropic hardening law and temperature-dependent degradation of elastic modulus and yield strength for post-irradiation Al6061 cladding. As temperature rises from 300 K to 600 K under 30 MPa internal pressure, concurrent mechanical degradation increases blister height by +20.0% and plastic strain by +21.6%, with the rate accelerating nonlinearly. Neglecting temperature-dependent elastic modulus overestimates the yield initiation pressure by up to 17.2% at 600 K, giving a non-conservative overestimation of the safety margin. Crack radius drives blister height in a near-exponential manner, whereas initial blister height has a limited effect. The modified framework proposed in this study provides a quantitative reference for the reliability assessment of plate-type fuel elements under irradiation and for blister failure prediction and safety evaluation of similar metallic claddings.

## 1. Introduction

Plate-type fuel elements are widely employed in research reactors because of their high power density and excellent thermal conductivity. Under irradiation, fission gases generated in the fuel meat [1,2] (i.e., the fuel-bearing region sandwiched between the cladding layers) gradually accumulate and can cause localized blistering of the cladding. Blisters not only alter the coolant-channel cross-section but may also lead to cladding rupture and release of radioactive products. Therefore, blistering is a critical failure mode that compromises fuel-element integrity. Except the mechanical integrity concern, blister-induced geometric deformation of the narrow coolant channels can significantly alter local thermal–hydraulic conditions. Recent experimental studies by Liu et al. [3,4] on bubble dynamics in narrow rectangular blistering channels revealed that the geometric structure of blisters affects bubble slide velocity and departure characteristics, further complicating the heat transfer behavior in the affected channels. The international Reduced Enrichment for Research and Test Reactors (RERTR) program has conducted extensive irradiation and annealing experiments to establish the relationship between blister-threshold temperature and burnup depth, providing a foundational database for fuel-element safety assessments [1,2,5,6,7].

From a physical perspective, blister evolution encompasses a complete chain of fission gas release, internal pressure buildup, cladding deformation, and eventual rupture. Early work can be traced back to the 1960s. Weir proposed a failure-analysis method for dispersion fuel elements based on plastic-yield criteria, using uranium fission burnup to predict component failure [8]. Gao et al. advanced a pore-connectivity mechanism for blister initiation spanning from nano- to macroscale, identifying three key stages by which individual pores coalesce into a blister [9]. In numerical modeling, Song et al. developed a quasi-static blister-growth model based on first-order shear deformation theory (FSDT) and Griffith crack-propagation criterion, coupling internal pressure with cap-shaped deformation to study the effects of thermal mismatch and plastic yielding, and validated their model against RERTR-12 experimental data [10,11,12]. In the relevant study, Song et al. [13] developed a multiscale mechanical model for U-Si/Al dispersion fuels under irradiation based on fission gas bubble evolution, predicting pore cracking behavior and circumferential stress distributions in the fuel meat. Zheng et al. simulated blistering failure of UMo–Al monolithic fuel elements under high-temperature, high-burnup conditions using ABAQUS and the extended finite-element method (XFEM), accounting for coupling between fission gas pressure and crack propagation, and obtained failure threshold temperatures, blister heights, and areas from RERTR-12 annealing tests [14]. For UMo/Zr monolithic fuel plates, Tang et al. established a numerical framework for blister evolution that couples thermal creep with internal pressure [15]. Additionally, Long et al. proposed a theoretical model for ceramic-particle failure in dispersion fuels considering matrix constraint, environmental factors, and particle size [16], while Chen et al. described metal-matrix crack propagation in dispersion systems using fracture mechanics [17], further enriching the simulation toolkit for fuel-element failure.

From an experimental perspective, the RERTR irradiation and annealing campaigns have provided systematic benchmark data on blister thresholds [1,2,5,6,7]. Wachs et al. reported blister-threshold temperatures for plates with various burnup depths in the RERTR-12 annealing series [2]; Meyer summarized key outcomes from the AFIP (ATR Full-size plates In center flux trap Position) and RERTR irradiation campaigns [18]. These experimental data serve as an essential basis for validating numerical models.

Except post-irradiation analysis and numerical modeling, fabrication and coating strategies have also been explored to enhance fuel-cladding interfacial integrity and mitigate fission-gas-related degradation. Jue et al. [19] characterized the microstructural features of HIP-bonded monolithic U-10Mo fuel plates with a Zr diffusion barrier between the fuel meat and Al6061 cladding, demonstrating that the HIP process effectively suppresses interdiffusion and maintains interface stability. Similarly, coating technologies have been investigated to improve cladding surface performance. Li et al. [20] conducted high-resolution TEM characterization of the TiN ceramic–metal interface on ferritic steels for nuclear applications, showing that the TiN diffusion barrier remains intact under irradiation up to 200 dpa and thermal cycling up to 550 °C, offering potential for mitigating fuel-cladding chemical interaction and blister-related coating failure. Getter materials represent another approach to mitigating fission-gas-related failure. Goto et al. [21] investigated ZrC coating as a free-oxygen getter in Pu-burner HTGR TRISO fuels, showing that ZrC reduces the partial pressure of CO gas generated from fission, thereby suppressing internal pressure buildup. While these getter and coating strategies have been primarily applied to other fuel systems, their underlying principles may offer insights for pressure management and interface design in plate-type fuel applications.

However, most existing models adopt simplified constitutive assumptions. Song et al. [10] used an elastic–perfectly plastic constitutive law without considering post-yield hardening. Moreover, the elastic modulus is frequently treated as constant, yet Kim et al. showed experimentally that the elastic modulus of Al6061 decreases nonlinearly with temperature above 423 K [22]. Ignoring this temperature dependence introduces errors in the elastic response and consequently affects the accuracy of predicted yield initiation pressures. The effects of irradiation on the mechanical properties of Al6061 are well documented. Farrell et al. reported that neutron irradiation significantly increases the yield strength of Al6061 by about 20% [23]. Farrell and King further showed that, for T6-tempered Al6061 after neutron irradiation, the 0.2% flow stress and ultimate tensile strength at 323 K and 373 K increased by 45–60% over the unirradiated values of approximately 280 MPa and 330 MPa, while elongation dropped from 15% to about 9%; at 423 K, similar hardening was observed but elongation further decreased to approximately 5% [24]. Farrell and Richt, in a study on High Flux Isotope Reactor (HFIR) hydraulic tubes, found that after irradiation, yield and ultimate stresses increased by 50–80% over the 55–200 °C range, while elongation decreased from 10–15% to about 5% at 55 °C and to about 3% above 100 °C [25]. Alexander [26], Gussev et al. [27] and Howard et al. [28] confirmed for aluminum that irradiation raises yield and ultimate strengths while reducing uniform and total elongations. Rest et al. compiled baseline material properties for Al6061, including elastic modulus, Poisson’s ratio, and fracture strength, providing reliable input for numerical simulations [29]. Xie et al. [30] systematically characterized fission product distribution in medium-burnup UO_2_ fuel using FIB-TEM techniques, revealing that intragranular Xe bubbles reach approximately 6.24 nm in diameter with a number density of 5.2 × 10^22^ m^−3^, providing experimental evidence for the morphological features of fission gas bubbles in irradiated fuels. Collectively, these experimental data indicate that the work-hardening capacity of irradiated 6061-T6 alloy is severely degraded, and its true mechanical behavior lies in the low-hardening end of the parameter space.

Based on the traditional elastic–perfectly plastic framework, this study introduces a bilinear isotropic hardening model and temperature-dependent degradation models for elastic modulus and yield strength. A systematic sensitivity analysis of the constitutive parameters is first conducted to establish a reasonable range and convergence conditions for the hardening parameters, thereby providing a reliable foundation for subsequent simulations. On this basis, the corrective effect of temperature-dependent mechanical property degradation on the elastoplastic blister response is quantitatively evaluated, and the differential effects of geometric parameters, specifically crack radius and initial blister height, on blister behavior are further investigated. The findings of this study provide a quantitative reference for the reliability assessment of plate-type fuel elements under irradiation and for blister failure prediction in similar metallic claddings.

## 2. Materials and Methods

### 2.1. Plate-Bending Model for a Blister

**Figure 1:**
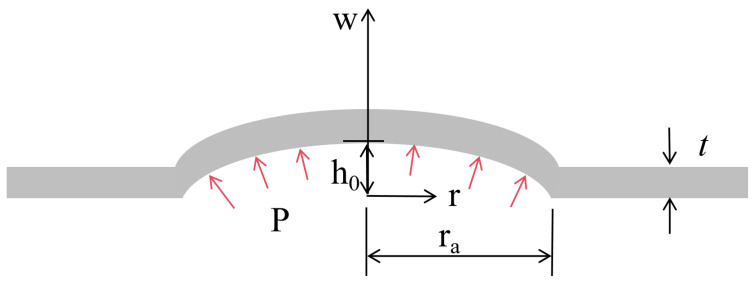
Schematic diagram of a pressurized blister.

**Figure 2:**
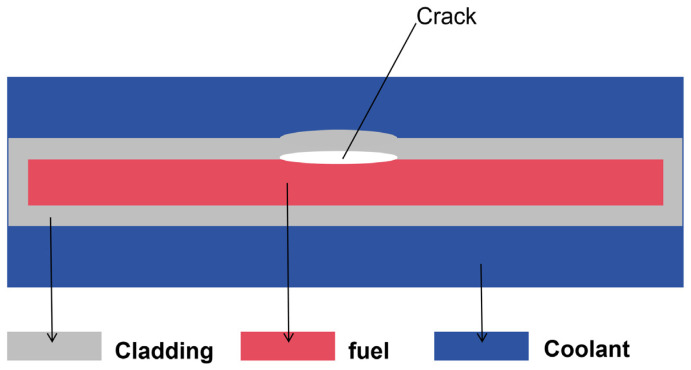
Schematic diagram of crack surface of monolithic fuel element.

The blister cap is simplified as a circular plate clamped at its periphery and subjected to a uniformly distributed internal pressure P, as shown in Figure 1 and Figure 2. The plate has radius r_a_, initial blister height h_0_, thickness t, elastic modulus E, and Poisson’s ratio ν. This idealization is justified by the axisymmetric nature of the blister region in fuel plates and has been widely adopted in both analytical and numerical studies of blister behavior.

When $t/r_{a}\ll 1$ and deflection w≪t, the classical thin-plate theory (CPT) gives the deflection equation:

(1) $$w\left(r\right)=\frac{P}{64D}{\left(r_{a}^{2}-r^{2}\right)}^{2}$$

where the bending stiffness D is defined as follows:

(2) $$D=Et^{3}/\left[12\left(1-\nu^{2}\right)\right]$$

The central maximum deflection h is as follows:

(3) $$h=Pr_{a}^{4}/\left(64D\right)$$

When $t/r_{a}$ is not negligible, transverse shear deformation contributes significantly. We adopt the first-order shear deformation theory (FSDT):

(4) $$w\left(r\right)=\frac{P}{64D}{\left(r_{a}^{2}-r^{2}\right)}^{2}+\frac{P}{4\kappa Gt}\left(r_{a}^{2}-r^{2}\right)$$

where G is the shear modulus and κ=5/6 is the shear correction factor. The FSDT analytical solution will serve as a reference for verifying the present finite-element model in Section 3.3. The central maximum deflection is then as follows:

(5) $$h=\frac{Pr_{a}^{4}}{64D}\left(1+\frac{r_{a}^{*2}}{r_{a}^{2}}\right)$$

(6) $$r_{a}^{*}={\left(\frac{24D}{\kappa Gt}\right)}^{1/2}$$

When $r_{a}\ll r_{a}^{*}$ (thick-plate regime), shear deformation dominates; when $r_{a}\gg r_{a}^{*}$ (thin-plate regime), Equation (5) reduces to the CPT solution [10,11,12].

### 2.2. Yield Criterion

The critical condition for the blister cap to enter plastic deformation under internal pressure is given by the von Mises yield criterion [31]:

(7) $$\sigma_{\mathrm{eq}}=\sqrt{\frac{1}{2}\left[{\left(\sigma_{1}-\sigma_{2}\right)}^{2}+{\left(\sigma_{2}-\sigma_{3}\right)}^{2}+{\left(\sigma_{3}-\sigma_{1}\right)}^{2}\right]}=\sigma_{Y}\left(T\right)$$

where $\sigma_{Y}\left(T\right)$ is the temperature-dependent yield strength. For a clamped circular plate, the maximum radial stress occurs at the lower surface at the plate edge ($r=r_{a}$) [32]:

(8) $$\sigma_{r,\max}=\frac{Pr_{a}^{2}}{2t^{2}}$$

This expression provides a rapid estimate of the yield initiation pressure. When $\sigma_{r,\max}\geq \sigma_{Y}\left(T\right)$, the blister cap yields and plastic strain begins to accumulate.

### 2.3. Blister Volume and Internal Pressure

Neglecting crack propagation, the blister cavity volume V is obtained by integrating the deflection profile [10]:

(9) $$V=2\pi \int_{0}^{r_{a}}w\left(r\right)r\;dr$$

Substituting Equation (4) gives the following:

(10) $$V=\frac{\pi Pr_{a}^{6}}{192D}+\frac{3\pi Pr_{a}^{4}}{16\kappa Gt}$$

If the fission gas behaves as an ideal gas, PV=NkT, the pressure and volume must be solved self-consistently by combining Equations (3) and (7). Song et al. [10,33] further provided simplified expressions for the total cavity volume $V_{tot}$ in the thick- and thin-plate limits:

For $r_{a}\ll r_{a}^{*}$ (thick plate),

(11) $$V_{tot}=\frac{1}{2}\pi r_{a}^{2}h$$

For $r_{a}\gg r_{a}^{*}$ (thin plate),

(12) $$V_{tot}=\frac{1}{3}\pi r_{a}^{2}h$$

The internal pressure levels adopted in this study are derived from the fission gas pressure model in Song et al. [10,33], where the blister cavity pressure is estimated by coupling the ideal gas law with blister volume evolution. In our simulations, these values are used as prescribed inputs rather than computed via an iterative coupling scheme.

### 2.4. Constitutive Model

#### 2.4.1. Temperature Dependence of Elastic Modulus

Kim et al. [22] conducted high-temperature tensile tests on Al6061 and found that the elastic modulus remains constant at E=68.60 GPa between 293 K and 423 K, but decreases as a quadratic polynomial above 423 K:

(13) $$E\left(T\right)=-38.28853+0.55362\;T-7.11488\times 10^{-4}\;T^{2}\;\left(\mathrm{GPa}\right),\;423\;K\leq T\leq 600\;K$$

#### 2.4.2. Temperature Dependence of Irradiated Yield Strength

The irradiation hardening of aluminum alloys originates from the evolution of microscopic defects. Farrell et al. [24] showed that neutron irradiation produces dislocation loops, transmutation silicon precipitates, and voids in Al6061, all of which impede dislocation motion and raise the yield strength. Based on dispersion-hardening theory (Orowan mechanism) [34], the strengthening increment from precipitates can be expressed as follows:

(14) $$\Delta \sigma_{\mathrm{disp}}=\frac{0.8MGb}{\lambda }ln\left(\frac{d}{b}\right)$$

where M is the Taylor factor (≈3.06), G the shear modulus, b the Burgers vector (0.286 nm for Al), λ the mean precipitate spacing, and d the mean precipitate diameter. Void hardening can be described in a similar form. The total post-irradiation yield strength can be approximated by linear superposition:

(15) $$\sigma_{Y}^{\mathrm{irr}}\left(T\right)=\sigma_{Y}^{0}\left(T\right)+\Delta \sigma_{\mathrm{disp}}+\Delta \sigma_{\mathrm{void}}$$

Equations (14) and (15) provide a micromechanical explanation for the irradiation-hardening effect. On this basis, Song et al. [10], using RERTR-12 post-irradiation annealing data, proposed a temperature-dependent fitting formula for irradiated Al6061 yield strength:

(16) $$\sigma_{Y}^{\mathrm{irr}}\left(T\right)=402.48-1.09\;T+0.0025\;T^{2}-2.16\times 10^{-6}\;T^{3}\;\left(\mathrm{MPa}\right),\;300\;K\leq T\leq 750\;K$$

**Figure 3:**
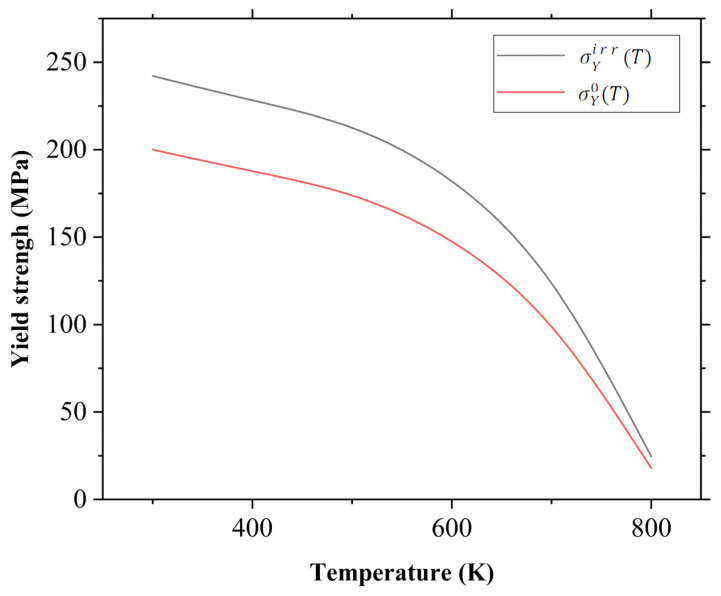
Temperature-dependence yield strength of irradiated Al6061 cladding Song et al. (2026) [10].

This formula adds approximately 20% irradiation hardening to the unirradiated baseline and has been validated against blister-threshold experiments. Figure 3 shows the yield strength of Al6061 in both irradiated and unirradiated conditions.

#### 2.4.3. Bilinear Hardening Model

For the plastic evolution driven by internal pressure in small blisters, a bilinear isotropic hardening model is adopted [35]. The model simplifies the stress–strain response into two linear stages: an elastic stage with slope E and a post-yield plastic stage with slope $E_{t}$ (the tangent modulus). The post-yield stress is given by the following:

(17) $$\sigma =\sigma_{Y}+E_{t}\;\varepsilon_{p},\;0\leq E_{t}\leq E$$

where $\sigma_{Y}$ is yield strength, and $\varepsilon_{p}$ is the equivalent plastic strain. $E_{t}=0$ corresponds to ideal plasticity (no hardening), while larger $E_{t}$ values indicate greater post-yield hardening capacity. By systematically varying $E_{t}$, the influence of hardening capacity on blister behavior is explored.

To link hardening capacity with dislocation dynamics, the Kocks–Mecking equation for dislocation density evolution can be invoked [36,37]:

(18) $$\frac{d\rho }{d\varepsilon_{p}}=k_{1}\sqrt{\rho }-k_{2}\rho$$

where $k_{1}$ is the dislocation storage coefficient and $k_{2}$ the dynamic recovery coefficient. The flow stress is related to dislocation density by the Taylor hardening equation [38]:

(19) $$\sigma =\sigma_{0}+\alpha MGb\sqrt{\rho }$$

where $\sigma_{0}$ is the lattice friction stress (initial yield stress) and α is an empirical constant (≈0.3). Combining with Equation (17), the tangent modulus can be written as follows:

(20) $$E_{t}=\frac{d\sigma }{d\varepsilon_{p}}=\frac{\alpha MGb}{2\sqrt{\rho }}\frac{d\rho }{d\varepsilon_{p}}$$

Equations (18)–(20) reveal the relationship between the macroscopic hardening parameter $E_{t}$ and the underlying dislocation evolution. In irradiated Al6061, the high density of defects suppresses dislocation storage (reduced $k_{1}$), resulting in significantly diminished hardening capacity; thus, the true material behavior lies in the low-hardening range ($E_{t}$ ≤ 2 GPa).

## 3. Numerical Model

### 3.1. Geometric Model

**Figure 4:**
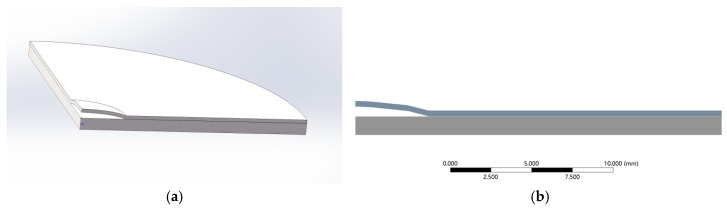
Schematic diagram of the geometric model: (**a**) overall assembly; (**b**) detailed blister region.

Following the axisymmetric modeling strategy of Song et al. [10], the blister structure is simplified as a cap-on-substrate assembly, as shown in Figure 4. The Al6061 cladding cap has thickness t = 0.35 mm, the substrate thickness is d = 1.15 mm, the pre-existing crack radius r_a_ is variable (baseline value 4.5 mm), and the initial blister height h_0_ is also variable (baseline value 0.5 mm). The substrate extends radially to R = 22.5 mm. A radial displacement constraint is applied at the symmetry axis.

### 3.2. Material Constitutive Parameters

The mechanical properties of irradiated T6-tempered Al6061 are based on the tensile data reported by Farrell et al. [24]. The elastic modulus follows Kim et al.’s temperature-dependent formula [22]: 68.6 GPa below 423 K, and the quadratic polynomial decline from 423 K to 600 K. The yield strength is described by Song et al.’s irradiated fitting formula [Equation (16)] over 300–750 K [10].

The bilinear isotropic hardening model is fully defined by three parameters: elastic modulus E, yield strength $\sigma_{Y}$, and tangent modulus $E_{t}$. To systematically assess the effect of hardening capacity, six values of $E_{t}$ are selected—0, 1, 2, 3, 5, and 7 GPa—covering the range from ideal plasticity to strong hardening. This range is chosen with reference to typical bilinear hardening parameters for aluminum alloys ($E_{t}$ ≈ 0.34~3.5 GPa, roughly 0.5–5% of the elastic modulus), with 5 GPa and 7 GPa included as upper-bound references. For subsequent temperature-softening and geometric-parameter analyses, $E_{t}$ = 2 GPa is adopted as the representative value. Based on the above analysis, the temperature-dependent material properties of irradiated Al6061 are obtained and applied in the subsequent finite-element simulations, as summarized in Table 1.

### 3.3. Mesh Convergence and Model Verification

**Figure 5:**
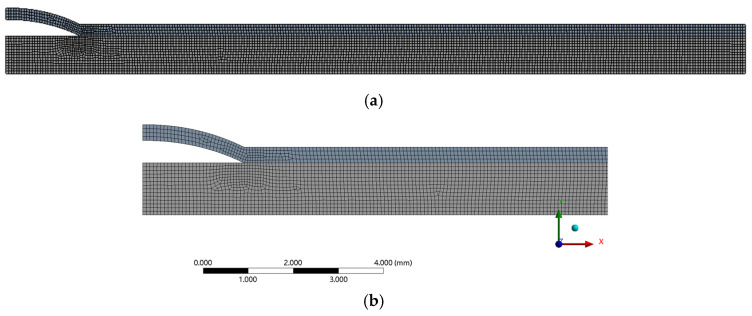
Finite-element mesh structure of the axisymmetric model: (**a**) overall diagram; (**b**) local magnified view.

**Figure 6:**
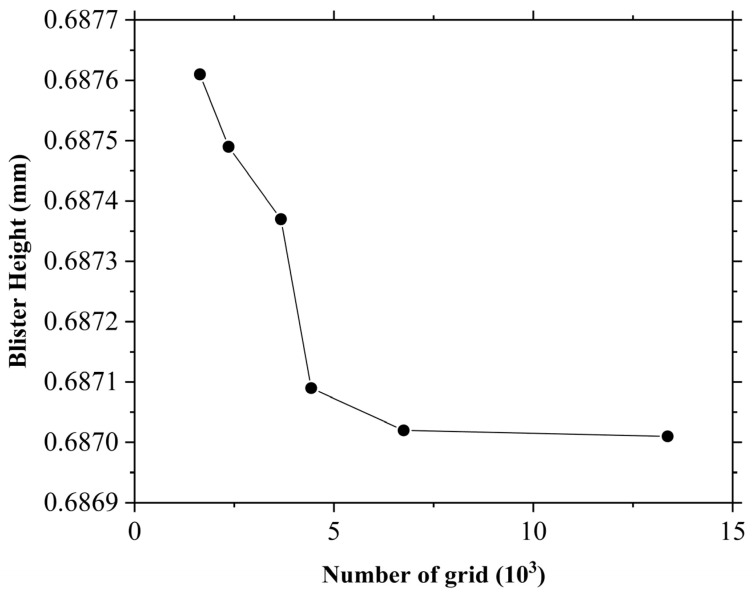
Mesh independence verification.

**Figure 7:**
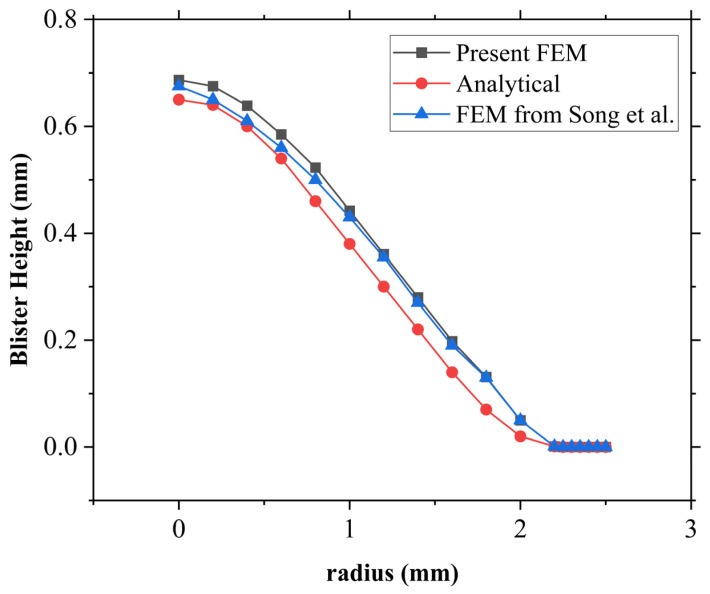
This comparison of elastic blister deflection profiles: analytical FSDT solution, present FEM, and Song et al. FEM results (P = 300 MPa, r_a_ = 2.25 mm) Song et al. (2026) [10].

The finite-element mesh uses CAX8R elements with local refinement at the crack tip and transitional elements in the substrate region (Figure 5). To verify mesh convergence and the overall finite-element (FE) modeling strategy, the elastic benchmark case of Song et al. [10] (P = 300 MPa, ra = 2.25 mm, purely elastic material) is used as a test condition. Six mesh densities with element counts of 1639, 2357, 3671, 4429, 6747, and 13,369 are evaluated. As shown in Figure 6, when the element count reaches 6747, further refinement changes the results by less than 1%; therefore, the 6747-element mesh is adopted for all subsequent calculations. The predicted blister deflection profile is compared with the FSDT analytical solution [Equation (4)] and with the finite-element method (FEM) results of Song et al. [10] (Figure 7). The central maximum deflection deviations among the three are all within 5%, demonstrating that the present FEM model accurately captures the elastic bending response and provides a reliable baseline for subsequent nonlinear analyses involving plasticity and temperature-dependent properties.

### 3.4. Sensitivity of Constitutive Parameters

To ensure numerical convergence and physical reasonableness in the subsequent temperature and geometric-parameter studies, a sensitivity analysis of the tangent modulus $E_{t}$ in the bilinear hardening model is performed. Six levels of $E_{t}$ (0, 1, 2, 3, 5, 7 GPa) are examined under T = 300 and internal pressures from 0 to 30 MPa.

**Figure 8:**
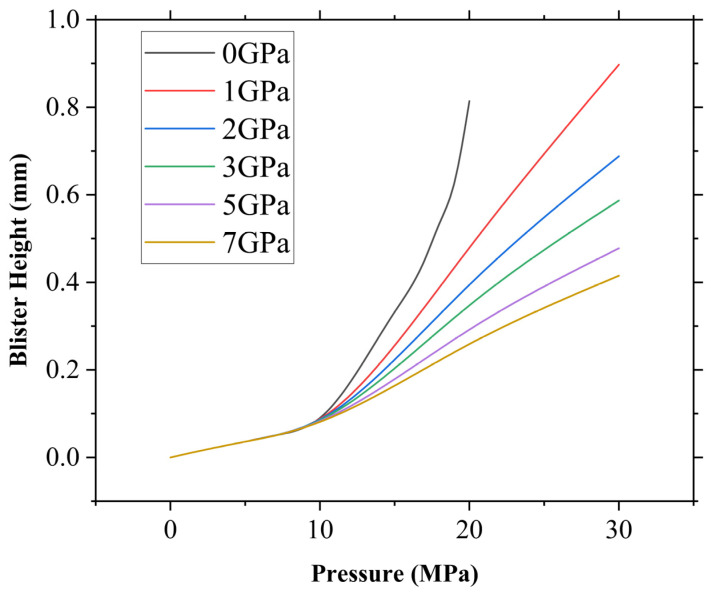
Blister height vs. internal pressure for different tangent modulus.

**Figure 9:**
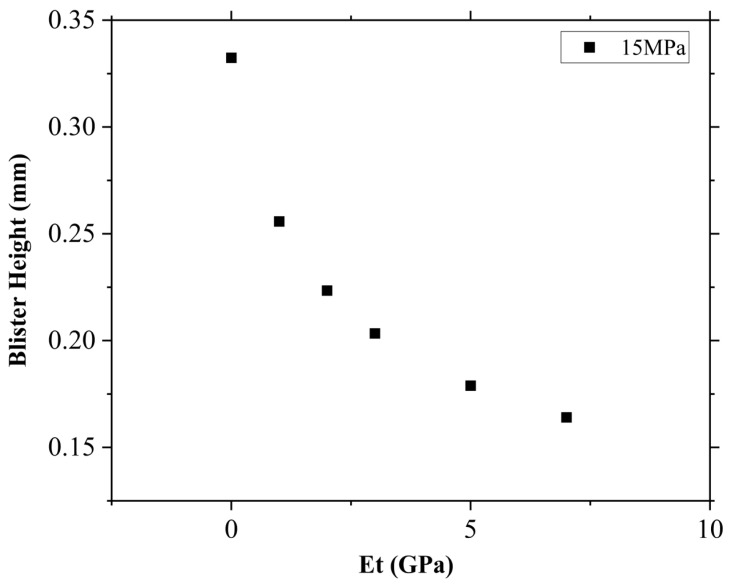
Marginal diminishing effect of hardening suppression.

Figure 8 shows the evolution of blister height with internal pressure for different tangent moduli. For $E_{t}$ = 0 (ideal plasticity), the numerical solution diverges above 20 MPa, indicating plastic instability in the absence of hardening. As $E_{t}$ increases, solution stability improves markedly. Table 2 summarizes the convergence behavior: for $E_{t}$ = 0 requires 500 substeps with stabilization and still converges slowly; for $E_{t}$ = 1 GPa, it requires a large number of substeps (200) with stabilization; and for $E_{t}$ ≥ 2 GPa, the solution converges smoothly with the default substep size (initial 50 steps) and without additional stabilization. Figure 9 further shows that the reduction in blister height is most pronounced when $E_{t}$ increases from 0 to 2 GPa; for $E_{t}$ ≥ 5 GPa, the curves flatten, indicating diminishing returns of hardening suppression.

Considering both numerical convergence and physical plausibility, and given that irradiated Al6061 exhibits a drastically reduced elongation and severely degraded work-hardening capacity [25,26], a tangent modulus of $E_{t}$ = 2 GPa (approximately 2.9% of the elastic modulus, 68.60 GPa) is adopted as the representative hardening parameter for the subsequent temperature and geometric parametric analyses. This value lies within the stable convergence regime and is consistent with the low-hardening characteristic of the irradiated material, thereby avoiding both the divergence risk associated with ideal plasticity and the over-hardening extreme.

### 3.5. Comparison with Previous Constitutive Models

To clearly highlight the constitutive refinements introduced in this work, Table 3 compares the key modeling features of the present study with those of previous studies that adopt a similar blister-growth framework [10]. While the overall modeling strategy follows the established approach in the literature, the present work introduces two substantive improvements at the constitutive level: (1) replacing the elastic–perfectly plastic assumption with a bilinear isotropic hardening model, with a systematic parametric study on the tangent modulus $E_{t}$; and (2) incorporating the temperature-dependent degradation of elastic modulus, which has been neglected in prior models but is essential for accurate high-temperature predictions.

## 4. Results and Discussion

It should be noted that all results in this section are obtained with the internal pressure treated as a prescribed boundary condition, as clarified in Section 2.3.

### 4.1. Effect of Temperature-Dependent Mechanical Properties on Blister Response

With $E_{t}$ = 2 GPa fixed, Kim et al. [22]’s temperature-dependent elastic modulus formula and Song et al. [10]’s irradiated yield-strength formula are simultaneously introduced into the FEM model to examine the blister response at five temperatures: 300 K, 400 K, 500 K, 550 K, and 600 K.

#### 4.1.1. Blister Height and Plastic Strain

**Figure 10:**
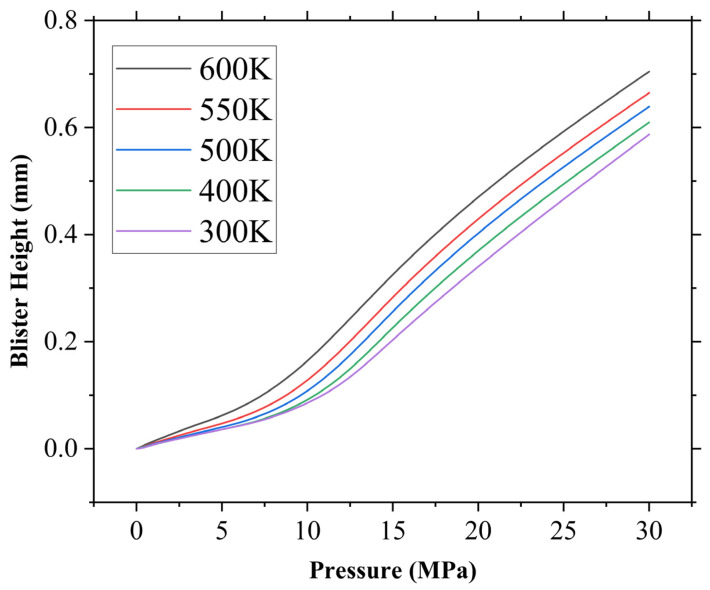
Blister height vs. internal pressure at different temperatures.

**Figure 11:**
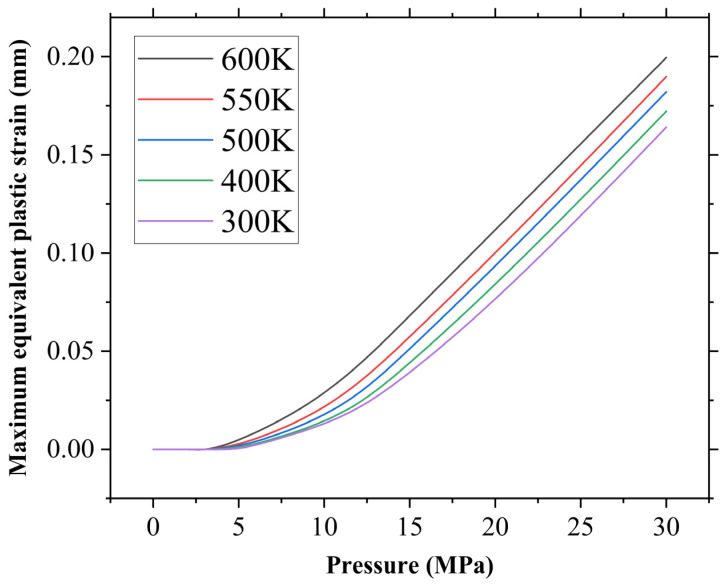
Maximum equivalent plastic strain vs. internal pressure at different temperatures.

Figure 10 and Figure 11 and Table 4 present the evolution of blister height (Height) and maximum equivalent plastic strain (PEEQ_max_) with internal pressure at different temperatures. Under an internal pressure of 30 MPa, as the temperature rises from 300 K to 600 K, the blister height increases from 0.59 mm to 0.70 mm, an increase of about 20.0%; the maximum equivalent plastic strain increases from 0.16 to 0.20 (+21.6%). This trend originates from the concurrent degradation of mechanical properties with temperature: the yield strength drops by about 24.9% (242 MPa → 182 MPa), facilitating plastic deformation, while the elastic modulus drops by about 45.0% (68.6 GPa → 37.8 GPa), increasing the elastic deflection under the same pressure. Their synergistic effect leads to a significantly enhanced blister response at elevated temperatures.

#### 4.1.2. Correction of Yield Initiation Pressure by Elastic Modulus Degradation

With temperature-dependent elastic modulus, the blister height–pressure curves separate already in the elastic stage. As shown in Figure 10, under an internal pressure of 10 MPa, the blister heights at 300 K and 600 K are 0.085 mm and 0.16 mm, respectively, with a difference of about 92.1%. This is because the reduced elastic modulus at high temperature produces a larger elastic deflection for the same pressure, an effect that cannot be captured with a constant elastic modulus.

**Figure 12:**
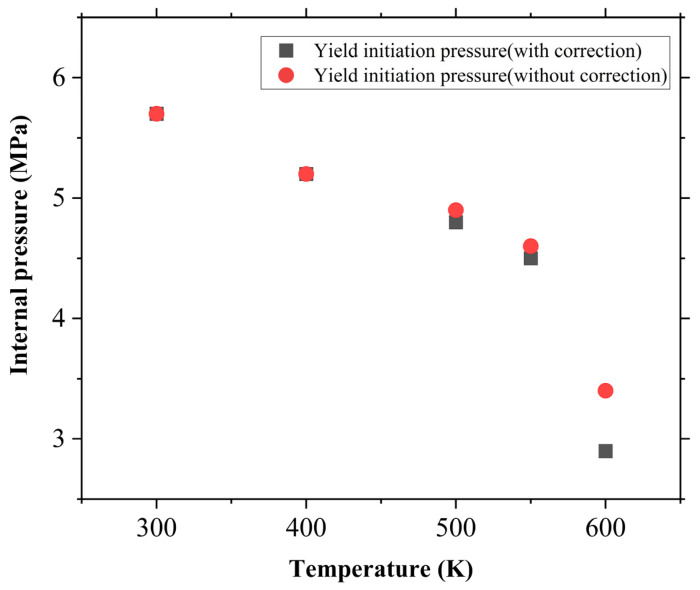
Yield initiation pressure at different temperatures.

Figure 12 and Table 5 compare the predicted yield initiation pressures using the “unmodified” elastic modulus (constant 68.6 GPa, neglecting temperature degradation) and the “modified” elastic modulus [Equation (13)]. At 500 K, neglecting elastic modulus degradation overestimates the initiation pressure by about 2.1%; at 600 K, the deviation expands to about 17.2%. These results demonstrate that, in high-temperature blister analysis, the elastic modulus must be treated as a temperature-dependent material parameter rather than approximated by its room-temperature constant value.

#### 4.1.3. Evolution of Plastic Zone with Temperature

**Figure 13:**
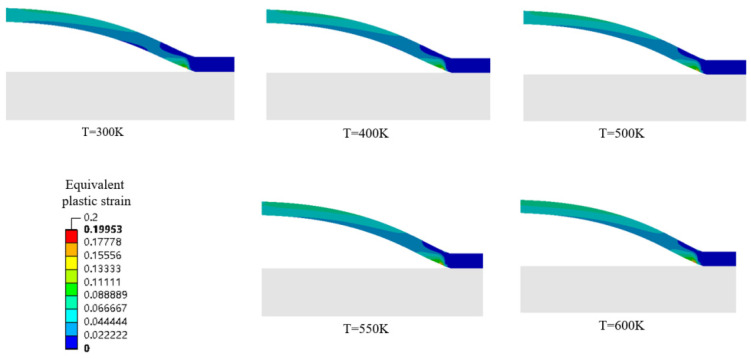
PEEQ contours under an internal pressure of 30 MPa for different temperatures.

Figure 13 shows the PEEQ contours under an internal pressure of 30 MPa for different temperatures. At 300 K, the plastic zone is confined to the vicinity of the crack tip; at 500 K, it expands to the central region of the blister cap; at 600 K, the entire blister cap is almost fully yielded. This progressive expansion from localized to global plasticity visually reflects the significant degradation of load-bearing capacity at elevated temperatures, which warrants attention in engineering assessments under high-temperature fuel-element operating conditions.

### 4.2. Differential Effects of Geometric Parameters on Blister Response

Under the modified constitutive framework ($E_{t}$ = 2 GPa, T = 300 K), the differential effects of crack radius and initial blister height on blister response are examined.

#### 4.2.1. Crack Radius

**Figure 14:**
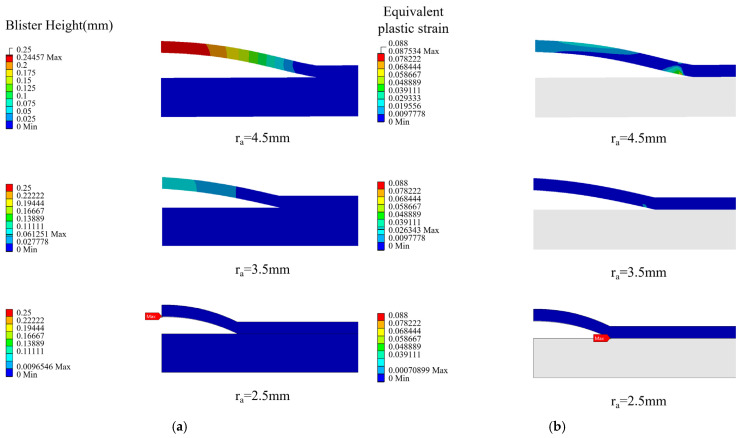
Finite-element simulation results of blister with crack radius of 4.5 mm, 3.5 mm, and 2.5 mm, respectively: (**a**) blister height contours; (**b**) PEEQ contours.

With initial blister height fixed at 0.5 mm and internal pressure at 15 MPa, the crack radius r_a_ is increased from 2.5 mm to 4.5 mm. Figure 14 and Table 6 show that the blister height increases dramatically from 0.0097 mm to 0.24 mm, a factor of approximately 25; the PEEQ_max_ increases from 7.1 × 10^−4^ to 8.8 × 10^−2^, a factor of about 123. The crack radius drives blister response in a superlinear manner and is the dominant geometric factor controlling the deformation amplitude within the investigated range.

From a mechanistic standpoint, increasing the crack radius affects two key aspects of the blister structure. First, according to thin-plate bending theory, under the same internal pressure and thickness, the central deflection scales with the fourth power of the radius [Equation (3)], so a small increase in radius leads to a power-law amplification of the elastic deflection. Second, a larger crack radius implies a longer clamped peripheral boundary, and the maximum radial stress [Equation (8)] scales with the square of the radius, promoting earlier plastic yielding and rapid expansion of the plastic zone toward the center. The coupling of these two effects produces an “elastic-baseline amplification + plastic-accelerated evolution” dual enhancement. Over the investigated range of 2.5 mm to 4.5 mm, each 1 mm increase in crack radius results in roughly an order-of-magnitude increment in blister height, exhibiting near-exponential rather than linear growth.

#### 4.2.2. Initial Blister Height

With crack radius fixed at 4.5 mm and internal pressure at 15 MPa, three initial blister heights are compared: h_0_ = 0.4, 0.5 mm, and 0.6 mm. As shown in Table 7, for h_0_ = 0.5 mm, the blister height is reduced by 13.4% compared with h_0_ = 0.4 mm; for h_0_ = 0.6 mm, the reduction is 25.4%. The initial blister height provides a pre-deformation that partially offsets the additional bending caused by internal pressure, but its influence is limited and does not alter the overall growth trend. Compared with the crack-radius effect, the initial blister height is a secondary factor.

## 5. Discussion

### 5.1. Parametric Origins and Model Scope

The input parameters adopted in this study are grounded in experimental and modeling data reported in the open literature. The temperature range of 300 K to 600 K is selected based on the blister-threshold temperatures documented in the RERTR irradiation and annealing campaigns [1,2,5,6,7], which cover the typical operational and off-normal thermal conditions of plate-type fuel elements. The internal pressure, ranging from 0 to 30 MPa, is derived from fission gas pressure estimation models for irradiated U-Mo fuels [10], representing a realistic loading level for post-irradiation blister growth. The crack radius range of 2.5 mm to 4.5 mm corresponds to typical blister dimensions observed in post-irradiation examination (PIE) of monolithic fuel plates [3,4,10,14], ensuring that the geometric parameters analyzed are within physically meaningful bounds. The temperature-dependent mechanical properties of Al6061 are taken from the experimental measurements of Kim et al. [22] and the irradiated fitting formula of Song et al. [10,24], respectively.

It is important to clarify the scope of the present model. This study focuses exclusively on the post-initiation blister growth phase, assuming that a blister cavity and a surface crack already exist at the fuel-cladding interface. The initiation mechanisms, namely, fission gas accumulation, bubble coalescence, and interfacial debonding, are not addressed. This scope is deliberately chosen to isolate and quantify the mechanical factors governing blister expansion once a blister has been formed, which is a prerequisite for predicting whether an existing blister will grow to a size that threatens cladding integrity. Rather than fitting a single experimental case, we perform parametric sensitivity analyses across a range of pressure, crack radius, and temperature values. This approach allows us to systematically map the blister response surface and identify the most influential parameters, providing more generalizable insights than a single case study.

### 5.2. Indirect Model Validation and Benchmarking

Direct quantitative validation against measured blister heights from PIE data is not yet available, as the present study focuses on the growth phase with idealized crack and cavity geometries. However, the model’s predictive capability is supported by three lines of indirect validation:

(1) Systematic mesh convergence tests (Section 3.3, Figure 6) confirm the numerical robustness of the finite-element implementation, with further refinement beyond 6747 elements changing the predicted blister height by less than 1%.
(2) Elastic deflection predictions show excellent agreement with the analytical FSDT solution [Equation (4)] and with the FEM results of Song et al. [10] under identical loading conditions (Figure 7), with central maximum deflection deviations within 5% across all three. This benchmark confirms that the present FE model accurately captures the elastic bending response of the blister cap.
(3) Parametric sensitivity results are qualitatively consistent with experimental observations reported in RERTR annealing studies [2,22], where blister dimensions are known to increase with temperature and burnup. The nonlinear acceleration of blister growth with rising temperature predicted by the present model mirrors the empirical trend that higher fission densities (and thus higher internal pressures) produce more pronounced blistering at elevated annealing temperatures. These benchmarks provide confidence that the model captures the essential mechanics of blister growth, even in the absence of case-specific experimental fitting.

### 5.3. Engineering Implications for Reactor Safety

Although research reactors typically operate with large safety margins against fuel failure, the present findings offer several practically relevant insights for engineering assessments.

(1) The 17.2% overestimation of yield initiation pressure at 600 K when neglecting temperature-dependent elastic modulus (Section 4.1.2, Table 5) reveals that even within the design safety envelope, material degradation can significantly shift the margin to yielding. This finding suggests that safety analyses relying on room-temperature or constant elastic modulus values may produce non-conservative estimates of the onset of plastic deformation in the cladding, particularly for high-temperature transients or localized hot spots.
(2) The near-exponential sensitivity of blister height-to-crack radius (Section 4.2.1, Figure 14) implies that once a critical crack dimension is reached, blister growth may accelerate rapidly. The behavior should be accounted for in inspection and threshold criteria. The quantitative relationship established in this study (each 1 mm increase in crack radius yields roughly an order-of-magnitude increment in blister height) provides a useful reference for defining acceptable defect sizes in fuel plate qualification.
(3) The parametric maps provided in this study (Figure 9, Figure 10, Figure 11 and Figure 12) can serve as a reference for evaluating hypothetical blister scenarios in safety assessments, especially for fuels that experience localized hot spots or irradiation-induced cracking. For instance, the temperature-dependent height and PEEQ curves (Figure 10 and Figure 11) allow engineers to estimate the expected deformation for a given combination of temperature and internal pressure, while the geometric sensitivity analysis (Figure 14, Table 6 and Table 7) quantifies the relative importance of crack size versus initial blister geometry.

Thus, while not intended as a direct predictive tool for a specific reactor, the present framework provides mechanistic understanding that can inform engineering judgment and, with future calibration, support the development of blister failure criteria for plate-type fuel elements.

### 5.4. Limitations and Future Work

Several limitations of the present study should be acknowledged to contextualize the findings and guide future research.

(1) The model assumes a pre-existing blister cavity and surface crack, and does not simulate the initiation mechanisms (e.g., fission gas bubble coalescence, interfacial decohesion, pore connectivity). The transition from incubation to growth remains outside the current scope. Future work will extend the framework to include initiation criteria, potentially through coupled damage-mechanics or phase-field approaches.
(2) The internal pressure is treated as a parametric input rather than being coupled to a fission gas release model. In reality, as the blister volume expands, the internal pressure decreases (for a fixed gas inventory) or increases (if additional gas is released from the fuel meat). This pressure–volume–temperature coupling is not yet implemented. Future work will incorporate gas-pressure evolution via the ideal gas law coupled with blister volume change, following the approach of Song et al. [10] but extended to account for temperature-dependent gas release kinetics.
(3) Direct experimental validation using PIE blister-height data or RERTR annealing test results is not yet performed, as such data are not readily available in the open literature for the specific Al6061 cladding materials considered here. While the indirect validation presented in Section 5.2 supports the model’s mechanical fidelity, quantitative comparisons with measured blister profiles remain a priority for future work.
(4) The temperature-dependent mechanical properties used in this study are based on data for irradiated Al6061 from multiple sources [10,22,23,24,25,26], but the combined dataset covers a limited temperature range (up to 600 K for elastic modulus and up to 750 K for yield strength). Extrapolation beyond these ranges should be undertaken with caution.
(5) The present model is specific to Al6061 cladding. Extension to other cladding materials would require re-calibration of the constitutive parameters using the corresponding high-temperature mechanical property data.

Despite these limitations, the parametric framework established in this study provides a mechanically rigorous basis for analyzing blister growth in irradiated metallic claddings. We prioritize the following directions for future research: (1) coupling with fission gas release and pressure–volume evolution models; (2) direct validation against PIE and annealing experimental data as they become available; (3) extension to other cladding materials (e.g., zirconium alloys); and (4) incorporation of thermal–hydraulic feedback to assess the coupled effects of blister-induced flow disturbance and cladding temperature evolution.

## 6. Conclusions

Through a comprehensive investigation of blister growth behavior in irradiated plate-type fuel element cladding, and based on the realistic mechanical property data for Al6061, this study systematically analyzes the effects of post-yield hardening capacity, temperature-dependent degradation of mechanical properties, and geometric parameters on the elastoplastic evolution of blisters. The main conclusions are drawn as follows:

(1) As temperature rises from 300 K to 600 K under 30 MPa internal pressure, concurrent mechanical degradation increases blister height by +20.0% and equivalent plastic strain by +21.6%, with the amplification effect accelerating nonlinearly at higher temperatures.
(2) Neglecting the temperature dependence of elastic modulus overestimates the yield initiation pressure at 600 K by approximately 17.2%, which would give a false sense of safety margin for high-temperature operations.
(3) Geometric analysis reveals that crack radius (2.5–4.5 mm) drives blister height in a near-exponential fashion and raises maximum equivalent plastic strain by over two orders of magnitude, whereas initial blister height exerts only a limited pre-deformation effect.

The above results indicate that crack radius and temperature-dependent mechanical degradation are the two dominant factors controlling blister failure, which should be prioritized in engineering monitoring and evaluation specifications. By substituting corresponding high-temperature mechanical property data, the proposed analysis framework can also provide a quantitative reference for the blister failure prediction of other metallic cladding materials.

## Figures and Tables

**Table 1:** Temperature-dependent material properties (irradiated Al6061).

Temperature (K)	Yield Strength (MPa)	Elastic Modulus (GPa)	Tangent Modulus (GPa)	Poisson’s Ratio
300	242.16	68.60	2.0	0.34
400	228.24	68.60	2.0	0.34
500	212.48	60.65	2.0	0.34
550	199.86	50.98	2.0	0.34
600	181.92	37.75	2.0	0.34

**Table 2:** Convergence comparison for different tangent modulus cases.

Eₜ (GPa)	Convergence Behavior	Required Initial Substeps	Stabilization Enabled
0	difficult	500	Yes
1	Poor	200	Yes
2	Good	50	No
3	Good	50	No
5	Good	50	No
7	Very good	40	No

**Table 3:** Comparison of constitutive modeling features between the present model and previous studies.

Feature/Modeling Aspect	Similar Past Models	Present Model	Improvement/Novelty
Constitutive model	Elastic-perfectly plastic	Bilinear isotropic hardening	Captures post-yield hardening behavior, providing more realistic plastic strain evolution.
Hardening parameter (tangent modulus $E_{t}$)	Not explicitly discussed or treated as a fixed value	Systematically investigated over a range (0–2 GPa)	Clarifies the influence of hardening capacity on blister response and identifies the low-hardening regime for irradiated Al6061.
Temperature dependence of elastic modulus	Not considered (constant E)	Included via temperature-dependent degradation model	Prevents overestimation of yield initiation pressure (up to 17.2% at 600 K), correcting a systematic bias in high-temperature predictions.
Temperature dependence of yield strength	Included	Included	Consistent with established experimental data, but now coupled with elastic modulus degradation.
Parametric sensitivity analysis	Limited or not performed	Systematic study on $E_{t}$, temperature, crack radius, and initial blister height	Quantifies the primary and secondary factors controlling blister growth (crack radius dominant, initial height secondary).

**Table 4:** Blister height and maximum equivalent plastic strain at different temperatures under an internal pressure of 30 MPa.

Temperature (K)	300	400	500	550	600
Height (mm)	0.59	0.61	0.64	0.66	0.70
PEEQ_max_ (mm)	0.16	0.17	0.18	0.19	0.20

**Table 5:** Comparison of yield initiation pressure with and without elastic modulus temperature correction.

Temperature (K)	Yield Initiation Pressures (Without Correction) (MPa)	Yield Initiation Pressures (with Correction) (MPa)
300	5.7	5.7
400	5.2	5.2
500	4.9	4.8
550	4.6	4.5
600	3.4	2.9

**Table 6:** Blister response under different crack radius (300 K, P = 15 MPa, h_0_ = 0.5 mm).

Crack Radius (mm)	Blister Height (mm)	PEEQ_max_ (mm)
2.5	0.0097	0.00071
3.5	0.061	0.026
4.5	0.24	0.088

**Table 7:** Blister response under different initial blister heights (300 K, P = 15 MPa, rₐ = 4.5 mm).

Initial Blister Height (mm)	Blister Height (mm)	PEEQ_max_ (mm)
0.4	0.28	0.031
0.5	0.24	0.088
0.6	0.22	0.044

## Data Availability

The original contributions presented in this study are included in the article. Further inquiries can be directed to the corresponding author.

## Abbreviations

The following abbreviations are used in this manuscript:

RERTR	Reduced Enrichment for Research and Test Reactors
ATR	Advanced Test Reactor
INL	Idaho National Laboratory
PIE	Post-irradiation examination
FSDT	First-Order Shear Deformation Theory
XFEM	Extended Finite-Element Method
AFIP	ATR Full-size plates In center flux trap Position
HFIR	High Flux Isotope Reactor
CPT	Classical Plate Theory
VCCT	Virtual Crack Closure Technique
FE	Finite Element
FEM	Finite-Element Method
PEEQ	Equivalent Plastic Strain
